# PCEP enhances IgA mucosal immune responses in mice following different immunization routes with influenza virus antigens

**DOI:** 10.1186/1476-8518-8-4

**Published:** 2010-08-24

**Authors:** Nelson F Eng, Srinivas Garlapati, Volker Gerdts, Lorne A Babiuk, George K Mutwiri

**Affiliations:** 1 Vaccine & Infectious Disease Organization/International Vaccine Center, University of Saskatchewan, 120 Veterinary Road, Saskatoon, Saskatchewan, S7N 5E3, Canada; 2 University of Alberta, 3-7 University Hall, Edmonton, Alberta, T6G 2J9, Canada

## Abstract

**Background:**

We previously demonstrated that polyphosphazenes, particularly PCEP, enhance immune responses in mice immunized subcutaneously and intranasally. The objective of the present study was to investigate the efficacy of polyphosphazenes as adjuvants when delivered through different routes of vaccine administration.

**Methods:**

BALB/c mice were immunized through intranasal, subcutaneous, oral and intrarectal delivery with vaccine formulations containing either influenza X:31 antigen alone or formulated in PCEP. Serum and mucosal washes were collected and assayed for antigen-specific antibody responses by ELISA, while splenocytes were assayed for antigen-specific cytokine production by ELISPOT.

**Results:**

Intranasal immunization with PCEP+X:31 induced significantly higher IgA titers in all mucosal secretions (lung, nasal, and vaginal) compared to the other routes. Serum analysis showed that all mice given the PCEP+X:31 combination showed evidence of enhanced IgG2a titers in all administered routes, indicating that PCEP can be effective as an adjuvant in enhancing systemic immune responses when delivered via different routes of administration.

**Conclusions:**

We conclude that PCEP is a potent and versatile mucosal adjuvant that can be administered in a variety of routes and effectively enhances systemic and local immune responses. Furthermore, intranasal immunization was found to be the best administration route for enhancing IgA titers, providing further evidence for the potential of PCEP as a mucosal adjuvant.

## Background

The high costs associated with the treatment of infectious diseases in humans or animals are a large financial burden. Thus, prevention of infections by means of vaccination remains the most cost-effective biomedical strategy. Since over 90% of infectious diseases are initiated by pathogens that traverse mucosal surfaces, stimulation of the mucosal immunity is the best approach to control such infections and this is best achieved through mucosal vaccination [1].

Mucosal vaccines need to induce immunity by at least one of three ways. They must prevent 1) the etiological agent from attachment and colonization at the mucosal epithelium, 2) replication and growth of the agent in the mucosa, and/or 3) toxins from attachment to their respective target cells [1]. As such, one of the primary determinants that would indicate enhanced mucosal immune response/protection is secretory IgA, the most abundant immunoglobulin found in human secretions. Secretory IgA is transported into mucosal secretions and is resistant to proteases, prevents adhesion of bacteria/toxins to target cells, and can neutralize viruses and toxins, among other characteristics [1]. Unfortunately, many mucosal vaccine candidates fail to stimulate a strong IgA immune response; as a result, only a very few approved human mucosal vaccines exist, such as Dukurol (cholera, oral route), and FluMist^® ^(influenza, intranasal) [1].

Mucosal administration of antigen without adjuvant often induces tolerance and fails to induce immunity. However, the addition of adjuvants to the antigen can break tolerance and lead to enhanced immune responses. Therefore, adjuvants are critical for the success of mucosal vaccines based on subunit antigens. Adjuvants that have shown to highly promote mucosal IgA and systemic IgG in mice include the cholera toxin (CT) and *E. coli *heat-labile enterotoxin (LT) [2,3]. However, their toxicities, even in genetically detoxified derivatives, make them unsuitable for human use. Other adjuvants, such as CpG oligodeoxynucleotides (ODN), can solely induce systemic and mucosal responses in mice; however, in larger animals, much higher doses of CpG are often required, which are not economically viable for use in livestock considering the cost of CpG ODN production [4]. As a result, CpG needs to be combined with other adjuvants to optimize its efficacy. Thus, there is a great need for safe and effective mucosal adjuvants. One class of adjuvants that has garnered attention in recent studies are polyphosphazenes. They are synthetic and biodegradable polymers that comprise a nitrogen and phosphosphorus backbone with organic side chains bound to phosphorus [5]. They can also be modified to include ionic groups which can increase solubility in water.

Polyphosphazenes such as poly[di(carboxylatophenoxy)phosphazene] (PCPP), have shown enhanced and long lasting immune responses with a variety of viral and bacterial antigens [6-10], including with influenza [5], tetanus toxoid, hepatitis B surface antigen (HBsAg), herpes simplex virus type 2 glycoprotein D [11], bovine respiratory syncytial virus [12] and non-microbial antigens such as bovine and porcine serum albumin [13,14]. Our previous studies showed that one of the newer polyphosphazene polyacids, poly[di(sodiumcarboxylatoethylphenoxy)phosphazene] (PCEP) has been shown to be more potent than PCPP in terms of quantity and quality of immune responses [13,15]. Also, PCEP was found to have long lasting [13], antigen-sparing effects [13], reduced the number of immunizations needed to induce similar immune responses from multiple immunizations with antigen alone and demonstrated mucosal adjuvant activity following IN delivery [15]. Cumulatively, these results demonstrate the potency of PCEP and raise the possibility of the development of a single-shot vaccine, which is highly sought not only as a cost-effective measure, but also to improve compliance with immunization schedules, particularly in developing countries.

Building upon this concept, an adjuvant that can be used in vaccine administered by a variety of routes would be highly desirable in the vaccine industry. However, with the exception of a few experimental adjuvants, many are not compatible with different routes of immunization. To further explore the versatility of PCEP, we investigated the adjuvant activity of PCEP with influenza X:31 antigen when administered by parenteral (subcutaneous), and mucosal (intranasal, oral, and intrarectal) routes. We show that, while PCEP has adjuvant activity in all routes tested, intranasal immunization was superior in elevating IgA mucosal immune responses and may be optimal for protection against influenza viruses.

## Methods

### Polymer synthesis and characterization

The polyphosphazene PCEP adjuvant was synthesized by Idaho National Laboratory (Idaho Falls, ID, USA) using methods described previously [9,16,17]. PCEP was found to have endotoxin levels below 0.034 ng/ml as assessed by the Limulus Amebocyte Lysate assay (Biowhittaker, Walkersville, MD, USA). The synthesized polyphosphazenes were in a solid salt state and dissolved in Dulbecco's PBS (Sigma, St. Louis, MO, USA) at a concentration of 5 mg/ml and used appropriately in vaccine formulations.

### Preparation of influenza virus X:31 antigen

Purified influenza X:31 virus (A/Aichi/68 H3:N2) was purchased from Charles River Laboratories (North Franklin, CT, USA). Briefly, the X:31 antigen was prepared from the virus stock by first diluting the virus with an equal volume of PBS, and then solubilised by adding and mixing Tween-80 to a final concentration of 0.25% at room temperature for 30 min. Subsequently, an equal volume of ether was added to the solution, and following another 30 min incubation with mixing, the solution was centrifuged to separate the non-soluble phases. The water-soluble phase was then collected and dried in a fume hood to evaporate residual ether for 1-2 days. The "split antigen" (X:31) was then quantified by the Quant-IT Protein Assay Kit (Invitrogen, OR, USA).

### Animals and immunization

All animal experiments were conducted according to the Guidelines for the Care and Use of Laboratory Animals as indicated by the Canadian Council on Animal Care and was approved by the Animal Care Committee of the University of Saskatchewan. BALB/c mice were obtained from Charles River Laboratories (North Franklin, CT, USA). PCEP was used at 50 μg per animal, and the experimental vaccine was formulated by mixing 5.0 μg of X:31 split antigen with an aqueous solution of PCEP.

A total of 8 random groups of BALB/c mice (n = 9 mice per group) were sedated and given a primary and a secondary immunization 4 weeks apart of either PCEP+X:31 or X:31 alone. Four groups of mice were given PCEP+X:31 through four different administration routes: intranasal (IN), subcutaneous (SC), oral, and intrarectal (IR) delivery (Table 1). The other 4 groups of mice were given X:31 only using the same above routes. Immunizations were given in 20 μl delivery (for intranasal vaccination, 10 μl per nostril), except for the oral route, which was given as a 50 μl volume. The immunization schedule is summarised in Table 1. Mice were bled prior to immunization (week 0) and subsequently at 2, 4, 6, and 8 weeks after the primary immunization. Any signs of adverse reactions to the immunizations were monitored. Vaginal washes were collected before immunization and at 4 and 8 weeks. After 8 weeks, nasal and lung washes were also collected and the spleens were dissected from all animals in order to assay the antigen-specific cytokines (IFN-γ and IL-4) in splenocytes.

**Table 1:** Immunization schedule.

Group	Vaccine formulation	Primary immunization	Secondary immunization (at 4 weeks after primary)
**1**	PCEP + X:31 EP	IN	IN
**2**	PCEP + X:31	SC	SC
**3**	PCEP + X:31	Oral	Oral
**4**	PCEP + X:31	IR	IR
**5**	X:31	IN	IN
**6**	X:31	SC	SC
**7**	X:31	Oral	Oral
**8**	X:31	IR	IR

IN, intranasal; SC, subcutaneous; IR, intrarectal

### Collection of mucosal washes

Lung, nasal, and vaginal washes were collected using a solution of the protease inhibitor Pefabloc SC^Plus ^(Roche, Indianapolis, IN, USA). Pefabloc powder was dissolved in PBSA and PSC protector solution as outlined by the manufacturer's instructions to a final concentration of 0.4 mM Pefabloc solution. For each wash, 100, 300, and 500 μl of Pefabloc solution was introduced into the vaginal, nasal, and lung cavities, respectively, and subsequently withdrawn for collection. All samples were promptly centrifuged and the resulting supernatants were collected and assayed for antigen-specific antibodies.

### Detection of influenza virus X:31- specific antibodies by ELISA

Procedures to assay for X:31-specific antibodies were followed as outlined previously [15] with minor modifications. Biotinylated goat-anti mouse IgG1 and IgG2a antibodies (Caltag Laboratories, CA, USA) were diluted 1/10000 to assay antibody titers in serum. IgG and IgA antibodies (Caltag) were used to analyze mucosal secretions. IgG antibodies was diluted 1/10000, while IgA was diluted to 1/5000. The sera from naïve, unimmunized mice were used as negative controls.

### Isolation of splenocytes

At the end of the 8 week experimental period, all of the animals were euthanized and spleens were removed similarly to previous experiments [15] with some modifications. Isolated spleens were placed in cold, complete MEM medium (Gibco, Carlesbad, CA, USA) containing 10 mM HEPES (Gibco) and 1X pen/strep antibiotics (Gibco). Cells were obtained by teasing spleen tissue with a syringe plunger through a 40 μm nylon cell strainer (BD Falcon, San Jose, CA, USA). Sterile NH_4_Cl was then added to the cell suspension to lyse erythrocytes for one minute and complete MEM medium was promptly added to prevent lysis of splenocytes. The splenocytes were washed once with complete MEM medium and resuspended in complete AIM V medium (10 mM HEPES, 1X non-essential amino acids (Gibco), 1 mM sodium pyruvate, 50 mM 2-mercaptoethanol) to a final concentration of 1 × 10^6 ^cells/ml. Cells were counted using a Multisizer™ 3 Coulter Counter (Beckman Coulter, Mississauga, ON, CA) according to the manufacturer's instructions. Cell concentrations were determined using software provided by the manufacturer.

### Detection of X:31-specific cytokine producing cells by ELISPOT

The protocol used to assay for X:31-specific IFN-γ and IL-4 producing cells similarly followed an approach previously described [15] with a few modifications. Rat anti-mouse IFN-γ and IL-4 (BD Biosciences, Mississauga, ON, CA) coated nitrocellulose microtiter plates (Whatman, Florham Park, NJ, USA) were instead washed and blocked with complete AIM V medium in a 37°C incubator. Splenocytes were added to these plates at 1 × 10^6 ^cells/well in complete AIM V medium in triplicate. Influenza X:31 antigen (1 μg/well) was added to appropriate wells containing the spleen cells and incubated at 37°C for 18 h. The ELISPOT assay and the counting of developed spots on the nitrocellulose plates were completed as previously described [15].

### Statistical analysis

All data on total IgG, IgG1, IgG2a, and IgA antibody titers in BALB/c mice were analyzed using GraphPad Prism version 5.01 for Windows, GraphPad Software, San Diego, California, USA, http://www.graphpad.com. The mean serum titers for ELISAs were examined for significance using repeated measures ANOVA with Tukey's Comparison of Rank Sum. Data from ELISPOT assays and ELISAs of mucosal secretions were examined using the Kruskal-Wallis test. If the means were found to be significant, median ranks between pairs of groups were performed using two-tailed Mann-Whitney U tests. Mean comparisons were conducted to compare the magnitude of responses. Significant effects were declared at p < 0.05.

## Results

### Antibody responses in serum of mice immunized using different routes of administration

The ability of a vaccine to enhance immune responses systemically is an important feature as infections can often spread. We previously determined that PCEP did not have an effect on its own in enhancing adaptive immune responses [15]. Hence, we did not include this control in the current studies. As such, to study the effect of PCEP as an adjuvant, PCEP+X:31 or X:31 alone were independently administered at four routes (IN, SC, oral, IR). Serum was collected and analyzed for antigen-specific antibodies.

**Figure 1:**
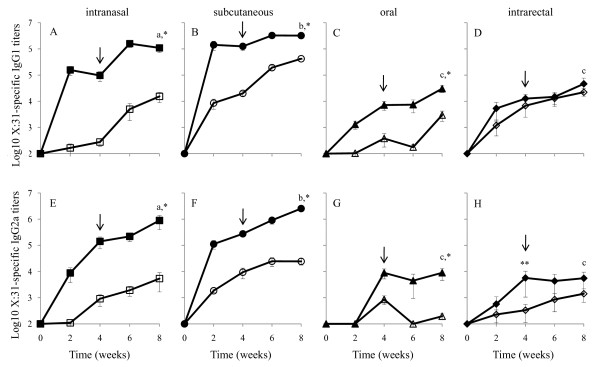
**PCEP enhances IgG1 and IgG2a X:31-specific serum titers when PCEP+X:31 is administered through intranasal, subcutaneous, and oral routes, but not after intrarectal immunization**. BALB/c mice were given PCEP+X:31 (closed symbols) or X:31 (open symbols) through IN (A, E, squares), SC (B, F, circles), oral (C, G, triangles) and IR (D, H, diamonds) immunization. X:31-specific IgG1 (A-D) and IgG2a (E-H) were assayed in mouse serum. Groups given PCEP+X:31 with different letters are significantly different from each other within each IgG subtype (p < 0.05). A single asterisk indicates an adjuvant effect of PCEP during the course of the experiment, while a double asterisk indicates an adjuvant effect only at the specified time point. Arrows indicate the time of delivery of a secondary immunization using the same delivery route as the primary immunization.

Following primary IN immunization with X:31 alone, there was very little induction of serum IgG1 titers up to 4 weeks post immunization (Fig. 1A, open squares). After the secondary IN immunization, IgG1 antigen-specific titers increased approximately 100-fold by the end of the 8 week experiment (Fig. 1A, open squares, p < 0.05). In contrast, IN immunization of PCEP+X:31 significantly enhanced IgG1 titers by approximately 1000-fold as early as 2 weeks after immunization (Fig. 1A, solid squares, p < 0.05) and remained steady for another 2 weeks. A secondary immunization with PCEP+X:31 further enhanced titers at least 10-fold at 6 weeks post primary immunization (Fig. 1A, solid squares). This observation confirmed that PCEP is a potent adjuvant for IN immunization either as a single or multiple immunization regimens.

Subcutaneous immunization with X:31 alone increased IgG1 titers by 100-fold as early as 2 weeks post immunization (Fig. 1B, open circles, p < 0.05) with little additional increase in titers at 4 weeks. The secondary immunization further enhanced titers by 10-fold at 6 weeks (Fig. 1B, open circles). Similar to IN immunization, SC immunization with PCEP+X:31 greatly increased the IgG1 response; after only 2 weeks, antigen-specific titers increased 10,000-fold (Fig. 1B, solid circles, p < 0.05). This response remained relatively consistent throughout the 8 week experiment, even after the second SC immunization (Fig. 1B). Thus, PCEP is a potent adjuvant for a primary SC immunization.

Generally, oral immunization (Fig. 1C) did not induce IgG1 titers as high as those seen from IN (Fig. 1A) or SC (Fig. 1B) immunization. Mice given X:31 alone orally showed very little serum IgG1 production from the first immunization and only after a secondary immunization, at the end of the 8 week experiment, did IgG1 titers increase approximately 10-fold (Fig. 1C, open triangles). When given PCEP+X:31, X:31-specific IgG1 titers were enhanced 10-fold at 2 weeks after primary immunization with a further 10-fold increase 2 weeks later (Fig. 1C, solid triangles). The secondary oral immunization with PCEP+X:31 did not further enhance IgG1 titers until 8 weeks, where titers slightly increased (Fig. 1C, solid triangles). Thus, PCEP has adjuvant activity when given by oral route.

Interestingly, the effect of PCEP was negligible when given by IR, since there was no difference in the serum IgG1 titers from mice given X:31 alone (Fig. 1D, open diamonds), or PCEP+X:31 (Fig. 1D, solid diamonds). IgG1 titers from either group initially showed increased titers at 2 weeks but did not show significant increase of titers following a second IR immunization (Fig. 1D). This observation suggests that PCEP has no adjuvant activity when given by IR route.

Comparing the four routes, it was clear that IN (Fig. 1A) and SC (Fig. 1B) delivery provided the highest titers of X:31-specific IgG1 titers (p < 0.05), particularly when PCEP was included in the formulations. Evidence that PCEP has an adjuvant effect was observed from IN, SC, and oral immunizations, particularly since enhanced IgG1 titers were seen as early as 2 weeks post immunization as seen for the three delivery routes (p < 0.05). PCEP did not show an adjuvant effect as serum IgG1 titers from mice given X:31 alone or PCEP+X:31 by IR were not significantly different.

The patterns of IgG1 responses seen were similarly observed when assaying for serum IgG2a X:31-specific titers (Fig. 1E-H). A primary IN immunization of X:31 alone in mice did not induce IgG2a titers until 4 weeks post immunization where a 10-fold increase was detected; a secondary IN immunization did not significantly enhance serum IgG2a titers (Fig. 1E, open squares). In contrast, mice given PCEP+X:31 showed a 100-fold increase of IgG2a titers at 2 weeks, followed by another 10-fold increase at 4 weeks after primary immunization (Fig. 1E, solid squares, p < 0.05). A secondary immunization at 4 weeks also did not significantly enhance IgG2a titers, although slight increases were observed at 6 and 8 weeks (Fig. 1E, solid squares).

Administration of X:31 alone by SC induced significant IgG2a titers at 2 and 4 weeks (Fig. 1F, open circles, p < 0.05). Similar to IN immunization of X:31, a secondary SC immunization did not significantly enhance serum IgG2a titers (Fig. 1F, open circles). The addition of PCEP to X:31 for SC immunization greatly enhanced IgG2a levels by 1000-fold as early as 2 weeks (Fig. 1F, solid circles). A secondary immunization at 4 weeks showed significant increases in IgG2a titers; however, the increases were not as large compared to titers observed from 2 weeks post immunization (Fig. 1F, solid circles, p < 0.05).

Assaying for serum IgG2a titers from mice given oral immunizations (Fig. 1G) also showed significant adjuvant effect of PCEP; however, the magnitude of those increases were not as large as compared to levels seen for IN and SC immunization, like the patterns observed for assaying IgG1. IgG2a induction was generally not observed in the serum of mice regardless of the number of X:31 immunizations except at 4 weeks where a slight 10-fold increase was observed, but diminished with the introduction of the second immunization (Fig. 1G, open triangles). The PCEP+X:31 formulation, however, showed a 100-fold increase of IgG2a titers at 4 weeks post immunization (Fig. 1G, solid triangles, p < 0.05). The increase in IgG2a titers persisted for the remainder of the 8 week experiment despite a secondary immunization (Fig. 1G, solid triangles).

As seen with IgG1 titers resulting from IR immunization, there was little difference between IgG2a titers in serum from mice given PCEP+X:31 or X:31 alone (Fig. 1H, solid and open diamonds, respectively), although IgG2a titers induced from PCEP+X:31 consistently showed slightly higher IgG2a titers during the course of the experiment (Fig. 1H, solid diamonds). Only at 4 weeks were IgG2a levels induced from PCEP+X:31 treatment significantly higher than titers induced from X:31 alone (Fig. 1H, double asterisk). While, the primary immunization appeared to increase IgG2a levels from either formulations, the secondary immunization with PCEP+X:31 or X:31 did not significantly enhance IgG2a titers (Fig. 1H).

Similar to the IgG1 assays, IgG2a titers were highest following IN (Fig. 1E) and SC (Fig. 1F) delivery, particularly in serum from mice that received PCEP+X:31 (p < 0.05). By the end of the 8 week experiment, serum IgG2a titers from IN and SC delivery were consistently at least 100-fold higher than oral or IR immunization. While the magnitude of the IgG2a titers was not as high in mice receiving oral immunizations, PCEP exhibited adjuvant activity (Fig. 1G, p < 0.05), as seen with IN and SC administration. IR immunization with PCEP+X:31 did not significantly induce higher IgG2a titers, except at 4 weeks (Fig. 1H, double asterisk).

Interestingly, PCEP appeared to have an ability to alter the quality of the immune response when delivered in most of the administration routes. Table 2 shows a comparison of IgG2a/IgG1 ratios calculated from the logarithmically transformed mean serum titers at 8 weeks post immunization as a way to evaluate whether Th1 or Th2 immune responses were being influenced by PCEP. Except for the IR route, IN, SC, and oral immunization with PCEP+X:31 greatly altered the IgG2a/IgG1 ratios compared to administration of X:31 alone. IN and SC immunizations nearly gave an IgG2a/IgG1 ratio of 1, indicating a balanced Th1/Th2 response, respectively, while the ratio following oral immunizations was elevated from 0.66 to 0.88 (Table 2). These results suggest that PCEP has a strong effect in enhancing X:31-specific IgG2a titers.

**Table 2:** Comparison of ratios of IgG2a to IgG1 in mice given either PCEP+X:31 or X:31 alone.

Formulation	X:31			PCEP+X:31		
**Delivery route**	**IgG2a**	**IgG1**	**IgG2a/IgG1 ratio**	**IgG2a**	**IgG1**	**IgG2a/IgG1 ratio**

Intranasal	3.73	4.19	0.89	5.95	6.04	0.99
Subcutaneous	4.39	5.62	0.78	6.41	6.51	0.98
Oral	2.29	3.47	0.66	3.95	4.48	0.88
Intrarectal	3.65	4.35	0.84	3.74	4.67	0.80

Mean serum titers at 8 weeks post-primary immunization were used for the calculations

### Comparing mucosal immune responses from different routes of delivery

**Figure 2:**
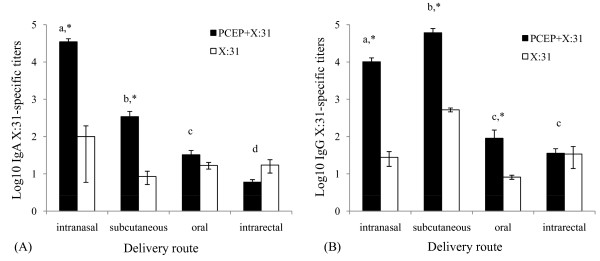
**PCEP enhances IgA and IgG antigen-specific titers in lung washes of mice following intranasal and subcutaneous immunization**. At the end of the 8 week experiment, lung washes from all the mice were collected and analyzed for X:31-specific IgA (A) and total IgG (B) titers by ELISA after receiving either PCEP+X:31 (closed bars) or X:31 alone (open bars) following IN, SC, oral, or IR immunization. Different letters indicate significant differences between groups that received PCEP+X:31, while asterisks indicate significant differences (i.e. adjuvant effect) between PCEP+X:31 compared to X:31 antigen alone (p < 0.05).

In order to determine the delivery route(s) which allowed for enhanced mucosal immune responses, we assayed antigen-specific IgA and IgG antibody titers in mucosal secretions of the lung, nasal, and vaginal cavities from all mice following IN, SC, oral, and IR immunizations. In lung washes, IN and SC immunizations showed the highest X:31-specific IgA titers induced by PCEP+X:31 (Fig 2A, solid bars, p < 0.05) and these were significantly higher than in mice immunized with X:31 alone (Fig. 2A, open bars, p < 0.05). However, mice given PCEP+X:31 by IN showed at least 100-fold more antigen specific IgA titers relative to SC immunization and a 1000-fold more IgA antibody titers compared to oral or IR immunizations (Fig. 2A, p < 0.05). Mice that received PCEP+X:31 by oral or IR routes showed very little IgA production (Fig. 2A). When assaying for total IgG in lung washes, IN, and SC immunizations of PCEP+X:31 induced the most total IgG titers compared to oral and IR delivery (Fig. 2B). Little response was observed from mice immunized orally or by IR relative to IN or SC (Fig. 2B). Although IN immunization did induce significant IgG production (p < 0.05), SC injection showed significantly more IgG antibody production in lung washes compared to mice immunized IN (p < 0.05). Significant adjuvant effect of PCEP in inducing IgG titers in lungs was observed for IN, SC, and oral immunization (Fig. 2B, p < 0.05).

**Figure 3:**
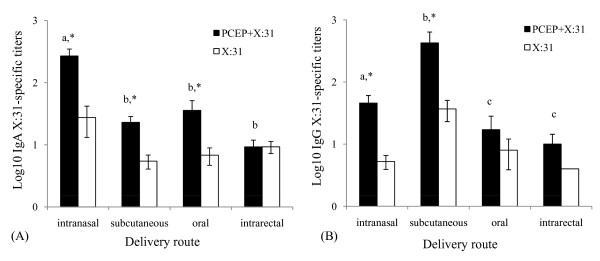
**Intranasal and subcutaneous immunization of PCEP + X:31 is effective in enhancing IgA and IgG titers in nasal secretions**. Nasal secretions were collected from mice receiving either PCEP+X:31 (closed bars) or X:31 (open bars) following IN, SC, oral, or IR immunization. Antigen-specific IgA (A) and IgG (B) was assayed from the nasal washes. Different letters indicate significant differences between groups that received PCEP+X:31, while asterisks indicate significant differences between PCEP+X:31 compared to X:31 antigen alone (p < 0.05).

The patterns observed from the mucosal immune responses in lung washes were similar to the responses assayed in nasal and vaginal mucosal secretions. Even though the magnitude of absolute titers were approximately 100-fold lower in nasal washes (Fig. 3) relative to lung washes (Fig. 2), IN immunization of PCEP+X:31 induced approximately ten fold more antigen-specific IgA titers compared to any other administration route (Fig. 3A, p < 0.05). Significantly higher nasal IgA titers were observed in mice that were given PCEP+X:31 by IN, SC, and oral immunizations compared to mice given X:31 alone (Fig. 3A, p < 0.05), while PCEP did not seem to have an effect on IgA titers in mice given formulations by IR. As in the lung washes, SC immunization of PCEP+X:31 showed greater antigen-specific total IgG antibody titers compared to IN immunization (Fig. 3B, p < 0.05). However, PCEP still had significant adjuvant activity following IN and SC immunizations (Fig. 3B, p < 0.05). Adjuvant activity was not observed in mice given formulations by oral or IR routes (Fig. 3B).

**Figure 4:**
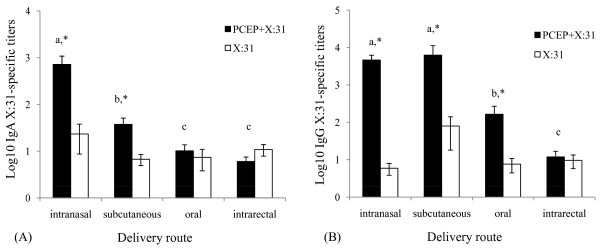
**Enhanced IgA and IgG titers from immunized mice are also found in vaginal secretions**. Vaginal mucosal washes were also collected at the end of the 8 week experiment where X:31-specific IgA (A) and IgG (B) was assayed from mice that were immunized by IN, SC, oral, or IR immunization with either PCEP+X:31 (closed bars) or X:31 alone (open bars). Different letters indicate significant differences between groups that received PCEP+X:31, while asterisks indicate significant differences between PCEP+X:31 compared to X:31 antigen alone (p < 0.05).

Analysis of vaginal secretions showed that IN immunizations of PCEP+X:31 led to significantly higher IgA titers compared to SC, oral or IR administration (Fig. 4A, solid bars, p < 0.05). Levels of IgA production induced by IN immunization of PCEP+X:31 were approximately 10 times more abundant than any other route of immunization (Fig. 4A). Similar to the IgA titers found in lung washes, only in IN and SC immunizations was the adjuvant activity of PCEP demonstrated (p < 0.05). Interestingly, while lung and nasal washes showed that SC immunization of PCEP+X:31 consistently induced higher total IgG antibody titers than IN (Fig. 2B, 3B), there were no significant differences between the total IgG from mice immunized by IN or SC (Fig. 4B). IgG titers induced by IN or SC delivery far surpassed levels detected in vaginal secretions found in mice immunized by oral and IR routes (Fig. 4B, p < 0.05). However, like IN and SC, oral immunization with PCEP+X:31 showed significantly higher IgG titers compared to antigen alone (p < 0.05).

### IFN-γ and IL-4 cytokine responses

**Figure 5:**
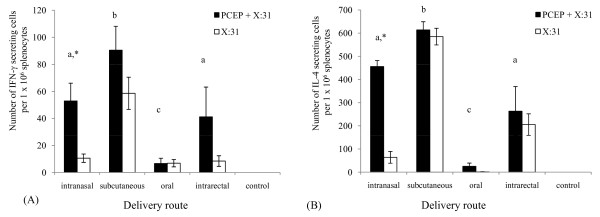
**PCEP significantly enhances IFN-g and IL-4 cytokine production in mice immunized via intranasal route**. The spleens from mice immunized with PCEP+X:31 (closed bars) or X:31 alone (open bars) were collected and splenocytes were subsequently isolated. The splenocytes were stimulated with X:31 antigen and antigen-specific IFN-γ (A) and IL-4 (B) were assayed to determine Th1/Th2 responses. Differences in cytokine production as a result of IN, SC, oral, or IR immunization were also examined. Different letters indicate significant differences between groups that received PCEP+X:31, while asterisks indicate significant differences between PCEP+X:31 compared to X:31 antigen alone (p < 0.05).

Mouse splenocytes were isolated and stimulated *in vitro *with X:31 antigen. Antigen-specific IFN-γ and IL-4 were assayed in culture supernatants in order to determine the ability of PCEP in enhancing cytokine production in T-helper (Th) cells in mice immunized by various routes. SC immunization with PCEP+X:31 demonstrated the highest frequency (or number) of IFN-γ and IL-4 cytokine secreting cells per 1 × 10^6 ^splenocytes (Fig. 5A, B, solid bars, p < 0.05) among the four routes of administration studied. An increase of the frequency of IFN-γ secreting cells was observed when PCEP+X:31 was delivered by IN, SC and IR. However, only IN immunizations induced significant numbers of IFN-γ secreting cells from mice given PCEP+X:31 (solid bars) compared to X:31 (white bars) alone (Fig. 5A, p < 0.05). IN immunization was also the route to induce significant IL-4 production (Fig. 5B, p < 0.05). Even though observations showed that oral administration of PCEP+X:31 led to significant increases in serum IgG1 and IgG2a antigen-specific titers (Fig. 1C and Fig. 1G), IgG in lung and vaginal washes (Fig. 2B, 4B), and IgA in nasal washes (Fig. 3A), it was interesting to note that very little IFN-γ or IL-4 producing cells were detected in splenocytes (Fig. 5A, B). Moreover, X:31 alone was able to induce high IL-4 production when delivered by SC, with nearly the same frequency as PCEP+X:31 delivered by the same route (Fig. 5B).

## Discussion

Most protein antigens are poorly immunologic when given mucosally and may induce immunological tolerance. Therefore, mucosal immunization with proteins requires the use of adjuvants to prevent this potential outcome. Many studies of polyphosphazenes have clearly showed that it can be a potent adjuvant to enhance the magnitude and alter the quality of immune responses [5,12-14,18,19]. However, many of these studies primarily examined the IN and SC routes. While previous studies showed that intramuscular (IM) and intradermal injection of PCPP and HBsAg was effective in enhancing total IgG titers in pigs [20,21], there have been no studies that have compared the adjuvant activity of polyphosphazenes when delivered by various mucosal sites. Since different mucosal surfaces have different microenvironments, mucosal adjuvants may well have different effects at different mucosal sites. For this reason, we evaluated the adjuvant activity of PCEP following vaccination via different mucosal routes (IN, oral, IR) in comparison to parenteral (SC) immunization.

This is the first investigation to study the effects of PCEP when co-delivered with influenza antigens following different immunization routes. The results suggest that PCEP is a versatile adjuvant as indicated by its adjuvant activity in all immunization routes tested (IN, SC, oral and IR). We also show that PCEP effectively enhances secretory IgA production, not only at the site of delivery, but also, through the common mucosal immune system, the effects can be observed at different and distal mucosal sites. Among the routes of administration studied here, IN delivery was clearly shown to be the most effective in enhancing influenza X:31-specific IgA production compared to other mucosal secretions from the nose, lungs, and vagina. Thus, IN immunization using PCEP as an adjuvant, may afford better protection against influenza virus infections compared to the other routes evaluated in the present studies.

Enhancing mucosal immune responses is a key indicator in determining whether polyphosphazenes have adjuvant activity at mucosal surfaces. Clearly, IN delivery of PCEP+X:31 was able to induce significantly higher antigen-specific IgA titers in all mucosal secretions tested compared to all other routes of delivery. The presence of secretory IgA in the mucosal secretions, particularly from IN immunizations, is a good predictor of protection against influenza infections, particularly since mucosal IgA in the upper respiratory tract have been found to be effective for the neutralization and clearance of influenza [22]. Previous studies have shown that PCEP enhances influenza virus neutralizing antibodies [13]. IN vaccination in mice has often shown to be the best route for protection against influenza challenge. Various live [23], inactivated [24-30], and subunit [31,32] influenza vaccine candidates have all shown that IgA, induced by IN delivery, is the primary contributor for protection against influenza, while strategies involving systemic or SC immunization either failed to induce IgA production [26] or afforded little protection [24,25]. In humans, secretory IgA responses have also shown to be elevated in the young and elderly given influenza vaccines by IN [33,34], although it is currently unknown if this relates to improved protection against influenza. It should be noted that IgG production is also important; while secretory IgA prevented viral-induced pathology in the upper respiratory tract, IgG was shown to be effective at neutralizing newly replicated virus after infection [23]. Thus, PCEP enhances both IgA and IgG, both of which play a major role in protection against influenza virus infection. Nonetheless, immunized mice still need to be challenged with live influenza virus to determine if actual protection is conferred.

Our results showed that while PCEP combined with influenza X:31 was not as potent following IR immunization, there was some evidence of adjuvant activity. IR vaccination is an effective strategy since lymphoid tissues in the rectum is a source of IgA precursors that are found in the genital tract [35]. In addition, immunization with hepatitis A [36] and *Mycobacterium *sp. [37] following IR delivery in mice afforded enhanced immune responses and protection. Since different antigens can have different effects on magnitude and quality of immune responses, it is likely that PCEP+X:31 may not have been an ideal vaccine formulation for IR immunization. Alternatively, high antigen doses may be required for IR immunization. However, since the versatility of polyphosphazenes showed adjuvant activity with a plethora of other antigens, it is possible that polyphosphazenes may have adjuvant activity with antigens such as hepatitis A or *Mycobacterium*.

Not surprisingly, immunization via IN was better than oral as the former may require less antigen and adjuvant than the latter. While the intestine has the greatest amount of lymphoid tissues, oral immunization presents significant challenges as the gastrointestinal tract is a very harsh environment for many antigens due to low pH in the stomach, digestive enzymes in intestines, and peristaltic movements. However, in this study, oral immunization of PCEP+X:31 induced significant antibody responses in sera and mucosal secretions, indicating that PCEP had potent adjuvant activity when administered orally. Previous studies showed that influenza antigens formulated in immunostimulating complexes (ISCOMs), did not increase serum antibody production after oral administration compared to immunization of antigen alone [38]. Also, this study is one of a few that uses the same amount of antigen and adjuvant for each delivery route tested. Many studies have often compared IN and oral immunizations using much more antigen for oral administration, where the amount of antigen used for oral delivery can range from 2.5 to 30-fold compared to IN delivery [39-42]. While we expected that immune responses following oral immunization would be lower in magnitude compared to IN, being able to demonstrate PCEP adjuvant activity in serum and mucosal secretions using a low dose antigen for this route of immunization is a remarkable achievement.

Not only did we show that PCEP has adjuvant activity, but also, the polyphosphazene seems to promote a more balanced Th1/Th2 responses based from antibody titers in serum (Table 2), as seen previously [15]. However, a correlation between antibody titers and cytokines responses in the current studies was not possible. A major factor was most likely the genetic background of BALB/c mice which seems to demonstrate a background expression of IL-4 not only in this study but also in previous ones as well [13,15], in contrast to other strains such as C57 BL6 [18].

While the concept of a mucosal vaccine affording more effective protection against pathogens that invade mucosal sites is ideal, previous studies clearly show two areas of concern when delivering antigens mucosally: 1) the ability of the antigen to retain itself and be taken up at mucosal surfaces is often ineffective [43] and as such, 2) leads to the difficulty in eliciting immune responses [44]. Although the mechanisms which mediate the adjuvant activity of polyphosphazenes are not known, the results from our studies would support the idea that the depot effect does not contribute to mucosal adjuvant activity of PCEP. Most mucosal adjuvants promote mucosal immunity by 1) acting through pattern recognization, like TLR, to stimulate epithelial cells, 2) enhancing antigen uptake through delivery and targeting, and 3) increase activation of APCs by upregulating costimulatory and MHC class II molecular expression, leading to increased interaction and thus, stimulation of effector B and T cells. As such, mucosal immunization remains very practical; highly trained personnel are not required, easier and less expensive to deliver, and no risk of needle injuries or syringe cross-contamination. Further benefits from such attributes also lead to improved compliance, which can be particularly important in developing regions of the world. As a result, the benefits of developing effective mucosal vaccines overcome the present barrier of challenges. Evolving strategies to improve antigen immunogenicity, delivery, and long-lasting effects for mucosal vaccines are constantly being developed.

Even though most studies of polyphosphazenes have been in mice, its adjuvant activity has been shown in rhesus monkeys [10], sheep [14], and pigs [20], without notable side effects. In addition, PCPP has been safely tested in Phase I clinical trials as an adjuvant with an influenza vaccine on young and elderly human adults with enhanced immune responses in sera and no side effects [45]. PCPP also been used in clinical trials on a HIV vaccine [46]. Clinical studies of PCEP have yet to be reported. Regardless, the use of polyphosphazenes in larger animals seems very promising as an effective and safe adjuvant, emphasizing its potential as an attractive adjuvant candidate for vaccine development.

## Conclusions

The polyphosphazene PCEP is a powerful mucosal adjuvant that can significantly enhance systemic and mucosal immune responses following immunization in a variety of routes. This study showed that polyphosphazenes delivered intranasally may be the ideal route of administration to enhance mucosal immunity or protection against influenza. We observed the potential of PCEP to enhance IgG2a and IgA levels, an observation seen previously with other adjuvants [47]. This suggests that the combined enhancement of cell mediated (IgG2a) and humoral (IgA) responses may be ideal for a cooperative effect in providing clearance and protection not only against influenza infections, but also possibly other respiratory pathogens, indicating its potential as an adjuvant for mucosal vaccines.

## Competing interests

The authors declare that they have no competing interests.

## Authors' contributions

NFE and SG conceived the study, design, and coordination of all of the mouse trials and assays. Additionally, NFE and GKM contributed to the primary preparation of this manuscript. VG and LAB were involved with critical analysis of the intellectual content. All authors read and approved the final manuscript.
